# Short-read and long-read RNA sequencing of mouse hematopoietic stem cells at bulk and single-cell levels

**DOI:** 10.1038/s41597-021-01078-4

**Published:** 2021-11-29

**Authors:** Xiuran Zheng, Dan Zhang, Mengying Xu, Wanqin Zeng, Ran Zhou, Yiming Zhang, Chao Tang, Li Chen, Lu Chen, Jing-wen Lin

**Affiliations:** 1 Key Laboratory of Birth Defects and Related Diseases of Women and Children of MOE, Department of Laboratory Medicine, State Key Laboratory of Biotherapy, West China Second University Hospital, Sichuan University, Chengdu, China; 2 Biosafety Laboratory of West China Hospital, Sichuan University, Chengdu, Sichuan 610041 China

**Keywords:** Stem-cell differentiation, Haematopoietic stem cells, RNA sequencing

## Abstract

Hematopoietic stem cells (HSCs) lie at the top of the differentiation hierarchy. Although HSC and their immediate downstream, multipotent progenitors (MPP) have full multilineage differentiation capacity, only long-term (LT-) HSC has the capacity of long-term self-renewal. The heterogeneity within the HSC population is gradually acknowledged with the development of single-cell RNA sequencing and lineage tracing technologies. Transcriptional and post-transcriptional regulations play important roles in controlling the differentiation and self-renewal capacity within HSC population. Here we report a dataset comprising short- and long-read RNA sequencing for mouse long- and short-term HSC and MPP at bulk and single-cell levels. We demonstrate that integrating short- and long-read sequencing can facilitate the identification and quantification of known and unannotated isoforms. Thus, this dataset provides a groundwork for comprehensive and comparative studies on transcriptional diversity and heterogeneity within different HSC cell types.

## Abstract (summary)

**Table:** 

Measurement(s)	short term hematopoietic stem cell • long term hematopoietic stem cell • multipotent stem cell
Technology Type(s)	RNA sequencing
Factor Type(s)	read length
Sample Characteristic - Organism	Mus musculus

Machine-accessible metadata file describing the reported data: 10.6084/m9.figshare.16759069

## Background & Summary

Hematopoiesis starts with a population of self-renewing hematopoietic stem cells (HSCs), which produce a series of increasingly more lineage-committed progenitor cells, ultimately giving rise to all types of mature blood cells. In the conventional model, long-term (LT) HCSs differentiates into short-term (ST) HSCs and subsequently multipotent progenitors (MPPs). Although all three populations have full multilineage differentiation capacity, they gradually lose self-renewal ability. Heterogeneity is presented in both HSC and MPP populations with distinct lineage bias^1^.

Both transcriptional and post-transcriptional regulations are pivotal in balancing the constitutive and low-level HSC turnover and downstream differentiation and hematopoietic reconstitution. In multicellular organisms, alternative splicing is a key post-transcriptional regulation mechanism that expands transcript diversity. Accumulating studies have revealed that alternative splicing patterns are essential during hematopoiesis. For instance, alternative splicing events specific in blood progenitors^2^ or megakaryocyte and erythrocyte lineage commitment^3^ were identified. Alternative splicing patterns in key hematopoietic regulators, such as HMGA2 was found to impact HSC molecular identity^4^. Moreover, aberrant AS is a hallmark of various types of cancer^5^, including leukemia^6^.

RNA sequencing (RNA-seq) that utilises short-read next-generation sequencing (NGS) or long-read sequencing (such as PacBio and Oxford Nanopore Technologies sequencing) is a powerful tool in understanding transcriptional diversity and regulation during various biological processes, including hematopoiesis^2^. While NGS is more reliable in expression quantification, the short reads can only provide limited information in AS events across the splicing joints. In contrast, the long-read sequencing methods offer a unique opportunity to examine alternative splicing isoforms with unprecedented accuracy in providing full-length information. Here we present comprehensive transcriptomic profiling of mouse HSC and MPP using both short-read and long-read RNA sequencing at bulk and single-cell levels.

## Methods

### Mice

C57BL/6 J mice were purchased from Beijing HFK Bioscience Co. Ltd (Beijing, China), housed and bred under SPF condition (Specific Pathogen Free) at Laboratory Animal Center of West China Second University Hospital and were allowed access to diet and water *ad libitum*. All animal experiments were carried out following the protocols approved by the Institutional Animal Care and Use Committee of West China Second University Hospital [(2018) Animal Ethics Approval No.004].

### Sample collection

**Fig. 1:**
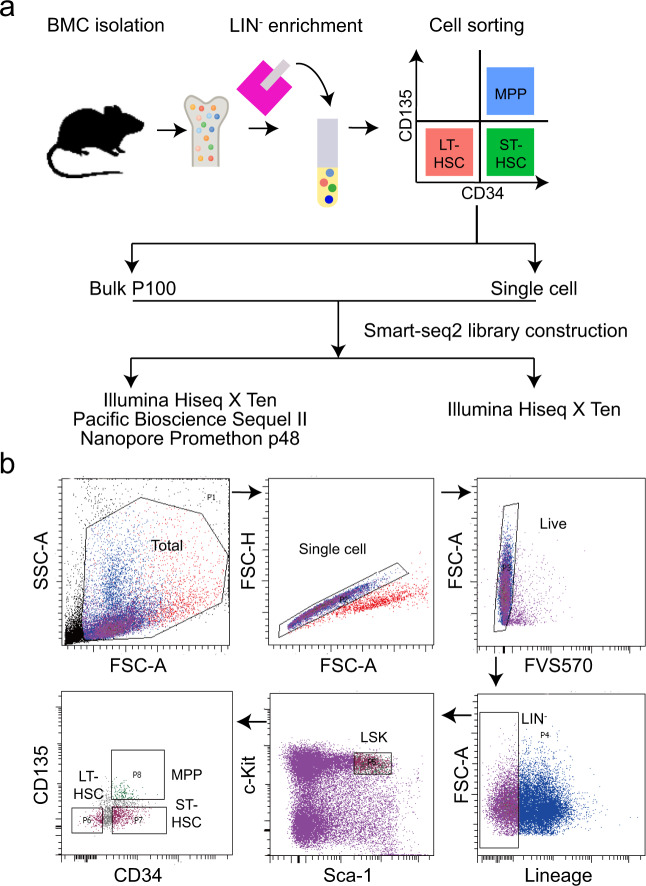
Sample collection and workflow. (**a**) Sample collection and sequencing methods. Bone marrow cells (BMC) were isolated, and lineage negative (LIN^−^) cells were enriched using magnetic beads. Long-term (LT) and short-term (ST) hematopoietic stem cells (HSC) and multipotent progenitors (MPP) were sorted according to their surface makers: LT-HSC (Sca-1^+^c-Kit^+^CD34^−^ CD135^−^), ST-HSC (Sca-1^+^ c-Kit^+^CD34^+^ CD135^−^) and MPP (Sca-1^+^ c-Kit^+^CD34^+^ CD135^+^). Single-cell or 100 cells (bulk P100) were sorted for library construction following the Smart-seq2 protocol. The cDNA libraries were used for short-read (Illumina Hiseq) or long-read (Nanopore or PacBio) sequencing. (**b**) Gating strategy for cell sorting. The main population was gated via FSC-A (forward scatter) and SSC-A (side scatter), and single cells were gated via FSC-A and FSC-H. FSC, forward scatter; SSC, side scatter; A, area; H, height.

The sample preparation and the overall workflow are summarized in Fig. 1. Female adult mice between 8-9 weeks were anesthetized and sacrificed by cervical dislocation. Bone marrow cells were isolated from the femur and tibia through a 70 μm cell strainer (BD Falcon). Hematopoietic stem and progenitor cells (HSPCs) were first enriched using EasySep™ Mouse Hematopoietic Cell Isolation Kit (STEMCELL, Cat No. 19856) with a lineage cocktail (biotinylated-CD11b, B220, Gr-1, TER-119 and CD3e) to remove mature cells, according to the manufacturer’s instructions.

To purify LT/ST-HSC and MPP, cells were sorted on a BD FACSAria SORP according to the following markers: LT-HSC [lineage (LIN)^−^Sca-1^+^c-Kit^+^CD34^−^CD135^−^], ST-HSC (LIN^−^Sca-1^+^c-Kit^+^CD34^+^CD135^−^) and MPP (LIN^−^Sca-1^+^c-Kit^+^CD34^+^CD135^+^). The following antibodies were used for staining (all purchased from BD): PerCP/Cyanine5.5 anti-CD11b Antibody (Clone M1/70, Cat No. 550993), PerCP/Cyanine5.5 anti-CD3e (Clone 145-2C11, Cat No. 551163), PerCP/Cyanine5.5 anti-TER119 (Clone TER119, Cat No. 560512), PerCP/Cyanine5.5 anti-Gr-1 (Clone RB6-8C5, Cat No. 552093), PerCP/Cyanine5.5 anti-B220 (Clone RA3-6B2, Cat No. 552771), BV421 anti-CD135 (Clone A2F10.1, Cat No. 562898), PE/Cyanine7 anti-Sca-1 (Clone D7, Cat No. 558162), APC/Cyanine7 anti-CD117 (c-Kit) (Clone 2B8, Cat No. 560185), FITC anti-CD34 (Clone RAM34, Cat No. 553733), Fixable Viability Stain 510 (BD Pharmingen, Cat No. 564406). Data were analyzed using FlowJoV10.7.1 software.

For single-cell RNA-sequencing (scRNA-seq), cells were individually sorted into 8-Strip PCR tubes containing the lysis buffer. For bulk RNA-seq, 100 cells (P100) were sorted into one PCR tube as a biological replicates. The batch information for cell sorting was included in Online-only Tables 1, 2.

### Library construction and sequencing

The cDNA libraries were generated following the Smart-seq2 protocol^7^ and the Illumina Nextera XT DNA preparation kit was used. The batch information of cDNA generation and sequencing was included in Online-only Tables 1-3.

#### Short-read sequencing

cDNA was then sheared randomly by Bioruptor Pico sonication device (Diagenode) for Illumina library preparation protocol including DNA fragmentation, end repairing, 3′ ends A-tailing, adapter ligation, PCR amplification and library validation. After library preparation, PerkinElmer LabChip® GX Touch and Step OnePlus™ Real-Time PCR System were introduced for library quality inspection. Qualified single-cell or P100 libraries were then loaded on Illumina Hiseq X Ten platform for PE150 sequencing. The average sequencing depth was 23.3 million reads (SD = 3.41) for P100 and 4.2 million reads (SD = 1.1) for single cells. Sequencing library construction and sequencing were done in Annoroad Gene Technology (Beijing, China).

#### PacBio sequencing

The full-length cDNA libraries were generated as described above, 5 µg of full-length cDNA were used for size selection using the BluePippin™ Size Selection System (Sage Science, Beverly, MA, USA). SMRTbell library was constructed using 1 μg size-selected (above 4 kb) cDNA with the Pacific Biosciences SMRTbell template prep kit. The binding of SMRTbell templates to polymerases was conducted using the Sequel II Binding Kit, and then primer annealing was performed. Sequencing was carried out on the Pacific Bioscience (PacBio) Sequel II platform. Sequencing library construction and sequencing were done in Annoroad Gene Technology (Beijing, China).

#### Oxford Nanopore Technologies cDNA sequencing

Similarly, the full-length cDNA was generated using the Smart-seq2 protocol as described above. About 250 ng cDNA were subjected to end-repair and dA-tailing ligation using NEBNext FFPE DNA Repair Mix (NEB, Cat No. M6630L) and NEBNext Ultra II End Repair/dA-Tailing Module (NEB, Cat No. E7645). PromethION library preparation was performed according to the manufacturer’s instructions (Oxford Nanopore Technologies, SQK-LSK109 Kit, EXP-NBD104 and EXP-NBD114). Sequencing was done on the Promethon p48 platform. Sequencing library construction and sequencing were done in Biomarker Technologies Co., Ltd (Beijing, China).

### Sequencing data analysis

All programs used default parameters unless stated otherwise in the corresponding section.

#### Single-cell short-read sequencing

Single-cell analysis was carried out using the Seurat R package (v3.2)^8^. We filtered cells using the following criteria: (a) feature counts below 500 or above 8000, (b) UMI counts below 70,000 or over 5,000,000, (c) identified as doublets using DoubletFinder (v2.0.3)^9^ with the parameter of “PCs = 1:6, pN = 0.25, pK = 0.09, nExp = 24”. A total of 109 LT-HSCs, 98 ST-HSCs and 107 MPPs were kept for further analyses. The quality statistics were provided in Online-only Table 1. We used principal component analysis (PCA) with variable genes as input and identified the top 6 significant PCs that were used as input for tSNE (t-distributed stochastic neighbor embedding). Cell markers from previously published studies^10,11^ were used to verify the cell types. Differentially expressed genes in the cell types were determined using the FindAllMarkers function in Seurat R package (v3.2)^8^.

#### Bulk short-read sequencing

The quality of the raw sequence data was analyzed using FastQC software (v0.11.8) (http://www.bioinformatics.babraham.ac.uk/projects/fastqc/) and RSeQC software^12^ (v3.0.1) (http://rseqc.sourceforge.net/). The paired-end short-reads were aligned to the mouse reference genome GRCm38 with annotation from ENSEMBL release 93 using STAR (v2.7.1a)^13^ with the parameters of “—outSJfilterOverhangMin 12 12 12 12 —alignSJoverhangMin 3 —alignSJDBoverhangMin 3 —chimSegmentMin 12 —chimScoreMin 2 —chimScoreSeparation 10 —chimJunctionOverhangMin 12 —outFilterMultimapNmax 1 —chimOutType Junctions SeparateSAMold”.

Gene body coverage, distribution of aligned reads over genome feature and RNA integrity at transcript-level were calculated using RSeQC^12^. The RNA integrity at the transcript-level was evaluated using the Transcript Integrity Number (TIN) algorithm^14^. These quality statistics were provided in Online-only Table 2. Gene expression was quantified using function summarizeOverlaps from GenomicAligments (v1.24.0)^15^ with parameters of “mode = “Union”, singleEnd = FALSE, ignore.strand = TRUE, fragments = TRUE”. The read counts were then normalized by function rlog with the parameter of “blind = FALSE” in DESeq2 (v1.28.1)^16^. Principal component analysis (PCA) and sample distances were calculated using FactoMineR (v2.4)^17^ and DESeq2 (v1.28.1)^16^, respectively. The batch effect (provided in Online-only Table 2) was corrected using ComBat from the sva package (v3.36.0)^18^.

#### Bulk long-read sequencing

The alignment was performed using Minimap2 (v2.17-r974-dirty)^19^ with the parameter of “*-ax splice*”. The number of reads, read length and quality were analyzed using NanoComp software (v1.33.1)^20^ with the parameter of “*–raw–store–tsv_stats*” and visualized using R package ggplot2 (v3.3.2)^21^ after filtering the reads with length below 200 or over 150,000 bp. These quality statistics were provided in Online-only Table 3.

Stringtie2 (v2.1.7) was used to assemble and quantify both short and long reads with or without a reference guiding. The parameter of the assembly process was “-p 20 -e -G -A”, and “-L” for long reads. The read counts of genes were extracted with a python script (prepDE.py3) with “-l 600” and normalized by function rlog with the parameter of “blind = FALSE” in DESeq2 (v1.28.1)^16^. The batch effects of gene expression from bulk Illumina, PacBio and Oxford Nanopore Technologies sequencing were corrected using ComBat from the sva package (v3.36.0)^18^. Principal component analysis (PCA) was calculated using plotPCA function in DESeq2 (v1.28.1)^16^. Correlation was calculated by function cor from R package stats (v4.0.2) with the parameter of “method = pearson”. GffCompare (v0.12.1)^22^ was used to compare and evaluate the accuracy of Stingtie2^23^ transcript assembly.

SUPPA2 (v2.3)^24^ was employed to classify the AS events with the parameter of “-f ioe -e SE SS MX RI FL”. Seven AS types were identified, including skipping exon (SE), retained intron (RI), alternative 5′ splice site (A5), alternative 3′ splice site (A3), mutually exclusive exons (MX), alternative first exon (AF), in which alternative first-exon use results in mRNA isoforms with distinct 5′ UTRs, and alternative last exon (AL), in which alternative use of multiple polyadenylation sites results in distinct terminal exons.

To visualize long-reads for transcripts, the gene regions were extracted using Samtools (v1.10.2)^25^. Bedtools (v2.30.0)^26^ was used to convert all bam files to GTF format. Visualization was performed using R package ggbio (v1.36.0)^27^. Sashimi plots of short-read sequencing data were plotted using pysashimi (https://github.com/ygidtu/pysashimi).

## Data Records

The raw data were deposited at NCBI BioProject under accession number PRJNA706066^28^. The accession number for individual samples were summarized in Online-only Tables 1-3. The processed files, including the quantifications of gene expression, isoform, splicing junction from both protocols were uploaded in figshare^29^.

## Technical Validation

### Quality control for short-read Illumina sequencing data

Read quality was assessed using FastQC, the number of total and clean reads, percentage of unique mapped reads, the mapped read length, Q30 and TIN (Transcript Integrity Number) statistics were provided in Online-only Tables 1-2. TIN indicates the RNA integrity at transcript-level^14^. The bulk RNA-seq samples generally have higher TIN scores than the single-cell samples. All single-cell samples had median TIN scores ranging from 24.8 to 51.8, with an average value of 40.7. Similar ranges of TIN were observed for all cell types (Online-only Table 1). Whereas the bulk samples had median TIN scores ranging from 49.4 to 66.1, with an average value of 56.9 (Online-only Table 2).

**Fig. 2:**
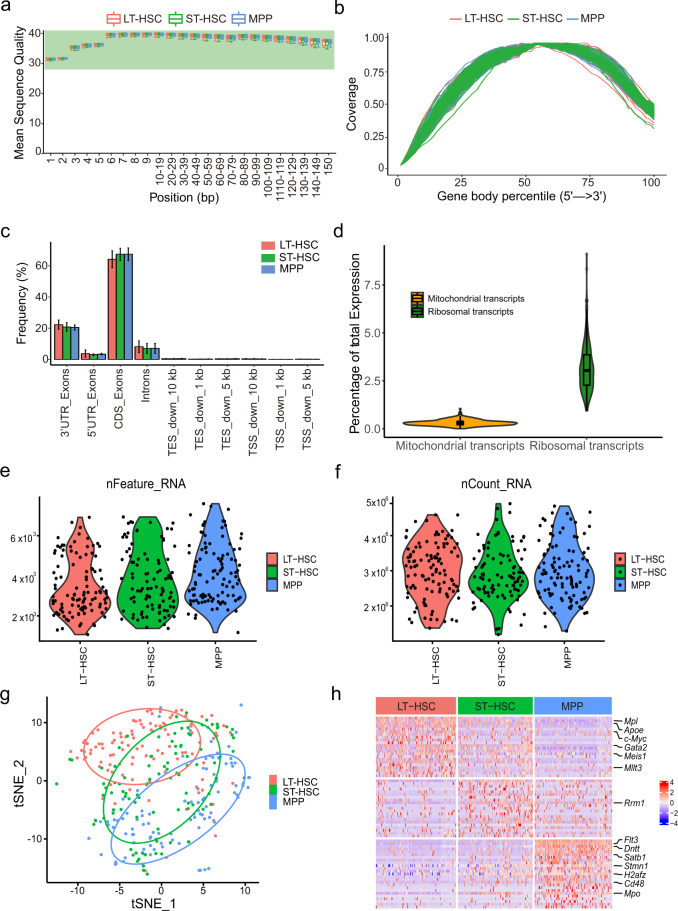
Quality check of single-cell Illumina short-read sequencing. (**a**) Boxplot showing average sequence quality per base for LT-HSC (red), ST-HSC (green) and MPP (blue) single-cell samples. (**b**) Gene body coverage of all detected genes. (**c**) Frequency of counts mapped in various gene regions. Bar chart showing mean and standard deviation. CDS, coding sequence; TES, transcription end site; TSS, transcription start site. (**d**) Violin plots showing percentage of reads that mapped to mitochondrial (orange) or ribosomal (green) transcripts. Violin plots showing the number of detected genes per cell (**e**) or the number of counts per barcode (**f**). (**g**) tSNE of three different cell types. (**h**) Heatmap showing the top 30 differentially expressed genes for each cell type (FDR < 0.05, log2(fold change) > = 1.5). The color scale indicates the expressing level.

**Fig. 3:**
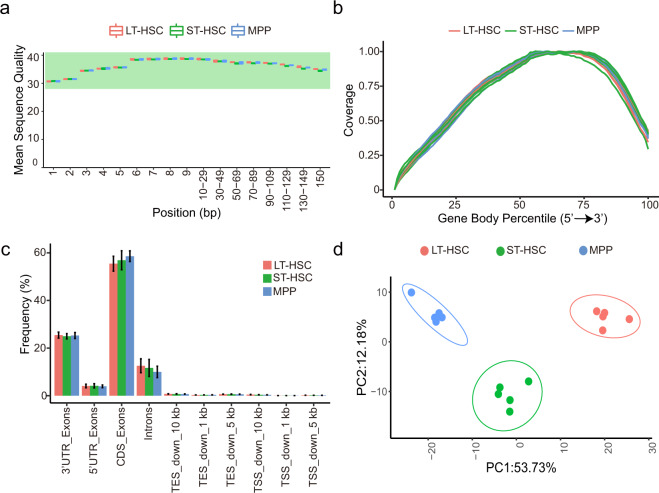
Quality check of bulk Illumina short-read sequencing. (**a**) Boxplot showing average sequence quality per base for LT-HSC (red), ST-HSC (green) and MPP (blue), 5 replicates per cell type. (**b**) Gene body coverage of all detected genes. (**c**) Frequency of counts mapped in various gene regions. Bar chart showing mean and standard deviation. CDS, coding sequence; TES, transcription end site; TSS, transcription start site. (**d**) Principal Component Analysis (PCA) of all samples after batch correction.

There was no significant difference in the distribution of average quality score per base among the samples of different cell types for both single-cell (Fig. 2a) and bulk (Fig. 3a) samples, and the reads were distributed approximately uniformly across the gene body for both datasets (Figs. 2b, 3b), indicating the high integrity of the RNA. We further checked the gene regions where the reads were mapped to and found that all samples have significantly more reads mapped to the exonic regions, while less were mapped to the intronic regions (Fig. 2c and Fig. 3c), in line with the observation of the previous reports^30^.

For single-cell sequencing data, we also examined the proportion of reads mapped to mitochondrial and ribosomal genes (Fig. 2d). The median percentage of mitochondrial genes per cell was 0.29 and the median percentage of ribosomal genes per cell was 3.04. MPP had the highest average number of detected genes (Fig. 2e), significantly higher than LT-HSC (p < 0.001, T-test), while the number of UMI counts per cell were comparable amongst three cell types (Fig. 2f). UMAP graph also showed that ST-HSC lied in between LT-HSC and MPP (Fig. 2g). The differentially expressed genes were analyzed between the cell types. There were 62, 63 and 266 differentially expressed genes (FDR < 0.05, log2(fold change) > = 1.5) in LT-HSC, ST-HSC and MPP, respectively. Furthermore, several known HSC signatures, including *Mpl, c-Myc, Mllt3, Gata2*, showed significantly higher expression in LT-HSC (Fig. 2h).

For bulk RNA sequencing, principal component analysis showed the three cell types separate on PC1 that accounts for 53.7% variation (Fig. 3d) after batch correction. Similar to the single-cell data, ST-HSC lied in between LT-HSC and MPP, corresponding to their biological status. Taken together, these results showed that the cells were correctly sorted and both the bulk and single-cell short-read RNA-seq data were of high quality.

### Quality control and concordance for long-read sequencing data

**Fig. 4:**
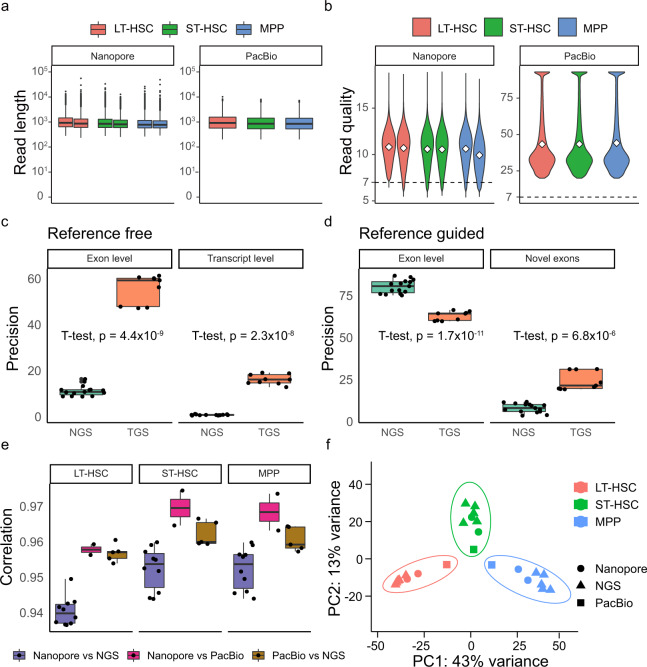
Quality check of bulk long-read sequencing. (**a**) Boxplots showing the length of reads per sample for nanopore (left) or PacBio (right) sequencing. LT-HSC (red), ST-HSC (green) and MPP (blue). (**b**) Violin plots showing read quality per sample for nanopore (left) or PacBio (right) sequencing. The white dots represent the means and the dashed line indicates the quality value of 7. (**c**) Precision of assembly accuracy at exon and transcript levels of Stingtie2 without a reference. (**d**) Precision of assembly accuracy at exon and novel exons levels of Stingtie2 with a reference. (**e**) Correlation coefficients of gene expression among the three sequencing methods. (**f**) Principal Component Analysis (PCA) of all samples from bulk Illumina, PacBio and Nanopore sequencing methods after batch correction.

The number of reads, read length, percentage of mapped reads and quality statistics were provided in Online-only Table 3. Nanopore sequencing data had a mean length of 1024 bp (Fig. 4a). Whereas PacBio sequencing data had the mean length of 946 bp (Fig. 4a). The quality score was higher for PacBio sequencing than nanopore sequencing (Fig. 4b), with an average of 47.57 and 10.53, respectively. The quality scores were comparable amongst all three cell types for both sequencing methods. Next, we compared the short- and long-read sequencing in terms of the precision of identifying exon and transcripts with or without reference. We found that long-read sequencing can provide relatively complete chains of exon including novel exons at transcript-level with or without reference (Fig. 4c,d), whereas short-read sequencing provides higher accuracy in defining exon junctions when a reference is available (Fig. 4d).

To assess the concordance between the replicates, we calculated the correlations of gene quantification between the short-read and long-read sequencing. The correlation coefficients were all over 0.93 (Fig. 4e), indicating high concordances among replicates. Moreover, the PCA illustrated that the samples were clustered by cell type between short-read and long-read sequencing replicates after batch correction (Fig. 4f). These results suggested that long-read sequencing data were of high quality, and the biological replicates are of high concordance. Moreover, the long-read sequencing enables the *de novo* identification and quantification of novel exon and transcripts.

### Overall alternative splicing patterns

**Fig. 5:**
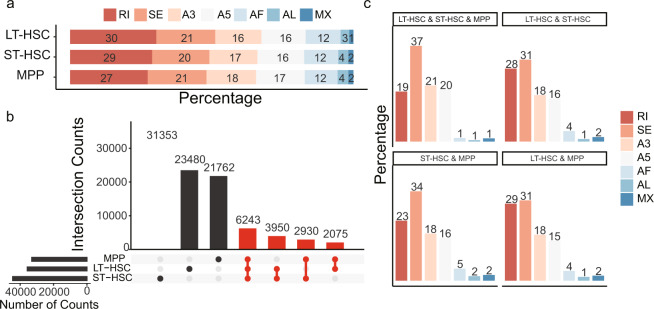
Overall patterns of alternative splicing and cell type-specific/shared events of bulk long-read sequencing. (**a**) Bar plot of the percentages of alternative splicing types in three cell types based on SUPPA2. (**b**) Bar plot showing the numbers of specific and shared alternative splicing events in three cell types. (**c**) Percentage of alternative splicing types for the shared alternative splicing events among cell types. SE, skipping exon; RI, retained intron; A5, alternative 5′ splice site; A3, alternative 3′ splice site; MX, mutually exclusive exons; AF, alternative first exon; AL alternative last exon.

To investigate the overall alternative splicing patterns using this long-read dataset, we first identified the alternative splicing events and types using SUPPA2^24^. Interestingly, the most prevalent type of alternative splicing was retained intron (RI) in all the cell types, followed by skipped exon (SE) and alternative 3′ or 5′ splice sites (Fig. 5a). Next, we revealed that more than 21,762 alternative splicing events were cell-type specific (Fig. 5b). The SE was the most-shared alternative splicing type among cell types (Fig. 5c), followed by RI and alternative 3′ or 5′ splice sites. These results suggested that the long-read sequencing facilitated the identification of large numbers of cell-specific or shared alternative splicing events that may be of potential function in hematopoiesis.

### Alternative splicing isoforms identification and quantification

**Fig. 6:**
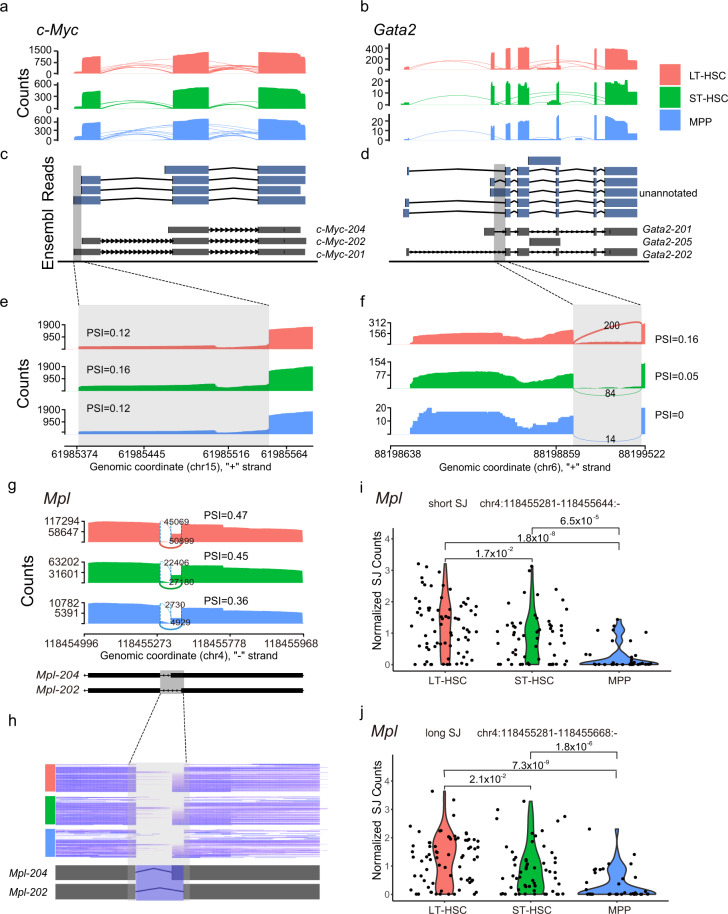
Alternative splicing isoforms identified using the long-read sequencing and quantified using the short-read sequencing. Sashimi plots showing long-reads mapped to c-*Myc* (**a**) or *Gata2* (**b**) in LT-HSC (red), ST-HSC (green) and MPP (blue) by Nanopore or PacBio sequencing. The identified nearly full-length transcripts (upper panel) and annotated transcripts in Ensembl (lower panel) for c-*Myc* (**c**) and *Gata2* (**d**) were shown. Sashimi plots showing Illumina generated short reads that are mapped to the indicated (shaded by grey) region for c-*Myc* (**e**) or *Gata2* (**f** ). (**g**) Sashimi plots of *Mpl* gene showing the decreased PSI of an alternative splicing event in LT-HSC (red), ST-HSC (green) and MPP (blue) by summing up the reads from all the single cells of each type. The percentage of spliced-in (PSI) for the indicated regions was calculated for each cell type. (**h**) Heatmap illustrates that the heterogeneity of alternative splicing of *Mpl* among single cells within each cell type. (**i**,**j**) Violin plots showing the normalized splicing junction counts of the short junction at chr4:118455281–118455644:- (**i**) and the long junction at chr4:118455281–118455668:- (**j**) in LT-HSC (red), ST-HSC (green) and MPP (blue).

To further confirm the quality of long-reads in identifying alternative splicing isoforms, we visualized the transcripts of known LT-HSC maker, *c-Myc* and *Gata2* (Fig. 2h) in the three cell types using nanopore and PacBio sequencing data. We screened all the reads mapped to the genes c-*Myc* and *Gata2* and their annotated transcripts. For c-*Myc*, we found 2915 reads, and LT-HSC had the highest number of reads (Fig. 6a). We visualized reads of full-length transcripts both in nanopore and PacBio sequencing and found that all annotated isoforms were identified (Fig. 6c). A 5′ alternative start site was found in the first exon of c-*Myc*. We used the short-read sequencing data to quantify the percentage of spliced in (PSI) for this locus and found that the longer isoform has a similar PSI (percent of splice-in) in all three cell types with ST-HSC had a slightly higher proportion of reads containing the longer isoform of the first exon (Fig. 6e). For *Gata2*, we found 595 reads, and similarly, LT-HSC had nearly 20 times more reads than the other two cell types (Fig. 6b). By comparing the full-length reads and annotated transcripts, we found an unannotated in Ensembl transcript with intron retention in LT-HSC (Fig. 6d). We further verified this intron retention using short-read sequencing data and quantified PSI for this intron with the highest PSI in LT-HSC (Fig. 6f). Next, we showed an example of integrating long-read sequencing with the single-cell RNA-seq based on Smart-seq2. We identified an alternative 5′ splicing site (A5) with a 24-bp alternative-spliced region in *Mpl* from our long-read sequencing data. Using our Smart-seq2 data, we identified that this event is differentially spliced with a decreasing PSI from LT-HSC (0.47), ST-HSC (0.45) to MPP (0.36) (Fig. 6g–j). The heterogeneity among single cells can be visualized using the Smart-seq2 data (Fig. 6h), and the involved long- and short-splicing junction (SJ) were significantly down-regulated during the hematopoiesis (Fig. 6i,j). These results indicated that integrating short- and long-read sequencing facilitates the identification of differentially regulated isoforms.

## Usage Notes

The bulk short-read RNA-seq can be used to quantify gene expression and alternative exon usage with high accuracy in various tissues or cell types. Single-cell RNA sequencing is powerful in revealing heterogeneity in gene expression within cell types. Full-length RNA-seq protocols such as Smart-seq2 also enable measurements of heterogenicity in alternative splicing. However, the assembly of transcripts using short-read sequencing remains difficult.

Our dataset provides a unique opportunity in revealing transcript diversities in the HSC population. By integrating the short- and long-read bulk sequencing datasets, one can better identify and quantify (novel) alternative splicing isoforms as shown above. The scRNA-seq data can further provide information on how these transcripts varied within different HSC cell types. Furthermore, this dataset is useful in developing statistical models to reconstruct the isoforms and enables further investigation of the largely unexplored post-transcriptional regulations such as alternative splicing and RNA editing at single-cell levels.

Gene expression and alternative splicing are regulated in gender-, age- and strain-specific manners, we generated data from female adult C57BL/6 J mice between 8-9 weeks in this study. Thus, users should take into consideration the gender, development stage and mouse strain when concluding. We expect that the accumulation of data will provide a more complete transcriptional and post-transcriptional landscape of hematopoiesis.

## Data Availability

The codes used in this article were deposited in https://github.com/LuChenLab/hemato.

## Online-only Tables

**Online-only Table 1:** Batch information and quality control for single-cell RNA-sequencing samples.

GEO Accession ID	Sample Name in GEO	cell sorting batch	cDNA preparation date	Qubit concentration (ng/ul)	cDNA peak (bp)	Sequencing lane	Cell type	Total reads (Million)	Uniquely mapped reads (%)	Average mapped length	Q30 (%)	GC (%)	TIN (median)	Inclusion
SRR13861999	LT-HSC6-1	20181211_batch1	20181212	4.3	1865.0	1	LT-HSC	6.1	86.3	297.9	91.6	50.0	41.2	yes
SRR13862043	LT-HSC6-2	20181211_batch1	20181212	9.1	1789.0	1	LT-HSC	6.8	82.3	297.6	91.1	48.0	41.2	no
SRR13861896	LT-HSC6-3	20181211_batch1	20181212	14.7	1854.0	1	LT-HSC	4.9	89.5	298.0	91.8	50.0	42.7	yes
SRR13862020	LT-HSC6-4	20181211_batch1	20181212	14.3	1873.0	1	LT-HSC	4.8	91.3	296.7	88.9	49.0	45.8	no
SRR13861943	LT-HSC6-5	20181211_batch1	20181212	3.0	1841.0	1	LT-HSC	5.1	87.7	297.8	90.0	49.5	36.4	yes
SRR13861886	LT-HSC6-6	20181211_batch1	20181212	18.7	1834.0	1	LT-HSC	4.8	89.2	297.8	91.2	49.0	39.8	yes
SRR13861874	LT-HSC6-7	20181211_batch1	20181212	13.3	1835.0	1	LT-HSC	6.6	83.0	298.2	92.6	49.0	33.1	no
SRR13861847	LT-HSC6-8	20181211_batch1	20181212	9.3	1823.0	1	LT-HSC	4.0	89.5	297.2	91.2	48.0	35.2	yes
SRR13861821	LT-HSC6-9	20181211_batch1	20181212	21.6	1848.0	1	LT-HSC	5.2	87.3	297.8	90.9	49.5	40.3	no
SRR13861786	LT-HSC6-10	20181211_batch1	20181212	19.5	1835.0	1	LT-HSC	5.8	92.2	297.7	91.0	49.0	45.9	no
SRR13862117	LT-HSC6-11	20181211_batch1	20181212	20.0	1836.0	4	LT-HSC	5.3	88.0	298.4	95.2	49.0	42.5	no
SRR13862054	LT-HSC6-12	20181211_batch1	20181212	7.4	1873.0	1	LT-HSC	3.8	69.5	298.3	92.7	51.0	36.6	yes
SRR13862050	LT-HSC6-13	20181211_batch1	20181212	10.6	1902.0	1	LT-HSC	4.3	90.3	296.4	91.2	47.0	41.7	no
SRR13862114	LT-HSC6-14	20181211_batch1	20181212	14.2	1892.0	1	LT-HSC	2.4	81.3	298.1	92.5	51.0	45.6	yes
SRR13862049	LT-HSC6-15	20181211_batch1	20181212	6.7	1862.0	2	LT-HSC	4.5	83.9	298.0	94.9	50.0	36.6	yes
SRR13862048	LT-HSC6-16	20181211_batch1	20181212	18.4	1858.0	2	LT-HSC	4.4	88.0	298.1	94.9	49.0	43.4	no
SRR13862047	LT-HSC6-17	20181211_batch1	20181212	18.0	1857.0	2	LT-HSC	5.0	88.5	298.0	95.0	49.0	37.2	no
SRR13862045	LT-HSC6-18	20181211_batch1	20181212	15.7	1860.0	2	LT-HSC	4.8	90.2	298.3	94.3	50.0	46.7	no
SRR13862044	LT-HSC6-19	20181211_batch1	20181212	11.7	1842.0	2	LT-HSC	4.2	83.0	298.3	94.5	50.0	38.3	yes
SRR13861907	LT-HSC6-20	20181211_batch1	20181212	12.9	1844.0	1	LT-HSC	2.6	82.8	298.0	92.5	48.0	30.5	yes
SRR13861906	LT-HSC6-21	20181211_batch1	20181212	15.3	1835.0	2	LT-HSC	4.7	88.2	298.4	94.7	50.0	45.2	yes
SRR13861904	LT-HSC6-22	20181211_batch1	20181212	10.4	954.0	1	LT-HSC	3.4	29.6	298.7	93.2	45.0	50.5	no
SRR13862046	LT-HSC6-23	20181211_batch1	20181212	16.1	1850.0	2	LT-HSC	4.6	87.8	298.5	94.7	50.0	43.5	yes
SRR13861903	LT-HSC6-24	20181211_batch1	20181212	20.6	1890.0	1	LT-HSC	2.6	85.1	298.2	92.4	50.0	44.2	no
SRR13861902	LT-HSC6-25	20181211_batch1	20181212	11.7	1856.0	2	LT-HSC	3.7	75.2	298.4	94.8	51.0	40.0	yes
SRR13861901	LT-HSC6-26	20181211_batch1	20181212	10.6	1848.0	3	LT-HSC	5.0	87.6	297.8	91.3	50.0	35.9	yes
SRR13861899	LT-HSC6-27	20181211_batch1	20181212	13.4	1833.0	3	LT-HSC	5.5	85.5	297.8	91.5	49.0	41.2	yes
SRR13861898	LT-HSC6-28	20181211_batch1	20181212	14.8	1830.0	3	LT-HSC	6.1	87.0	297.8	91.6	49.0	42.4	yes
SRR13861897	LT-HSC6-29	20181211_batch1	20181212	13.6	1862.0	1	LT-HSC	3.2	86.6	297.9	92.6	50.0	39.8	yes
SRR13861895	LT-HSC6-30	20181211_batch1	20181212	15.2	1830.0	3	LT-HSC	2.8	85.5	298.1	92.1	49.0	41.7	yes
SRR13861893	LT-HSC6-31	20181211_batch1	20181212	15.6	1910.0	3	LT-HSC	6.3	83.8	297.7	91.4	50.0	44.6	yes
SRR13861900	LT-HSC6-32	20181211_batch1	20181212	15.1	1910.0	3	LT-HSC	5.4	88.3	297.5	91.2	49.0	40.2	yes
SRR13861892	LT-HSC6-33	20181211_batch1	20181212	18.2	1893.0	3	LT-HSC	5.4	82.6	297.8	91.4	50.0	42.9	yes
SRR13861891	LT-HSC6-34	20181211_batch1	20181212	15.7	1896.0	3	LT-HSC	6.4	87.8	297.6	91.1	50.0	40.3	yes
SRR13862026	LT-HSC6-35	20181211_batch1	20181212	17.5	1900.0	3	LT-HSC	5.7	82.0	297.7	91.5	50.0	42.4	yes
SRR13862024	LT-HSC6-36	20181211_batch1	20181212	17.7	1879.0	4	LT-HSC	2.6	87.7	298.5	95.5	49.0	39.7	yes
SRR13862023	LT-HSC6-37	20181211_batch1	20181212	16.2	1877.0	4	LT-HSC	4.6	74.5	298.4	95.5	50.0	27.8	yes
SRR13862022	LT-HSC6-38	20181211_batch1	20181212	9.0	1898.0	1	LT-HSC	3.6	89.4	297.0	92.5	48.0	39.2	yes
SRR13862021	LT-HSC6-39	20181218_batch1	20181219	8.2	1879.0	4	LT-HSC	4.7	87.8	296.4	94.3	49.0	46.3	no
SRR13862018	LT-HSC6-40	20181218_batch1	20181219	4.7	1844.0	1	LT-HSC	2.1	88.6	296.2	91.7	49.0	40.3	yes
SRR13862025	LT-HSC6-41	20181218_batch1	20181219	8.9	1886.0	1	LT-HSC	2.3	92.4	297.5	92.5	49.0	43.0	yes
SRR13862017	LT-HSC6-42	20181218_batch1	20181219	11.2	1928.0	4	LT-HSC	3.6	88.6	297.7	95.4	49.0	40.5	yes
SRR13862016	LT-HSC6-43	20181218_batch1	20181219	15.3	1895.0	1	LT-HSC	3.8	89.9	297.7	92.5	47.0	39.3	yes
SRR13862015	LT-HSC6-44	20181218_batch1	20181219	11.5	1900.0	1	LT-HSC	2.5	89.1	297.7	92.6	47.0	35.4	yes
SRR13862014	LT-HSC6-45	20181218_batch1	20181219	11.6	1912.0	4	LT-HSC	3.0	88.9	297.2	94.5	49.0	45.7	yes
SRR13862013	LT-HSC6-46	20181218_batch2	20181219	8.8	1883.0	1	LT-HSC	2.2	88.4	297.6	93.2	47.0	34.9	yes
SRR13862012	LT-HSC6-47	20181218_batch2	20181219	15.7	1879.0	4	LT-HSC	5.2	90.3	297.9	95.1	47.0	38.2	yes
SRR13862011	LT-HSC6-48	20181218_batch2	20181219	7.2	1867.0	4	LT-HSC	4.8	87.1	297.0	94.7	48.5	37.8	yes
SRR13862010	LT-HSC6-49	20181218_batch2	20181219	9.3	1890.0	1	LT-HSC	2.7	88.2	296.5	92.0	48.0	39.5	yes
SRR13861942	LT-HSC6-50	20181218_batch2	20181219	12.6	1876.0	1	LT-HSC	2.8	89.8	297.6	92.7	47.0	40.1	yes
SRR13861941	LT-HSC6-51	20181218_batch2	20181219	9.3	1716.0	1	LT-HSC	2.5	91.4	297.6	92.5	48.0	34.9	yes
SRR13861940	LT-HSC6-52	20181218_batch2	20181219	7.6	1827.0	1	LT-HSC	6.2	85.2	296.7	92.3	48.0	30.2	yes
SRR13861939	LT-HSC6-53	20181218_batch2	20181219	10.8	1858.0	1	LT-HSC	2.4	88.0	297.3	92.3	48.0	32.8	yes
SRR13861938	LT-HSC6-54	20181218_batch2	20181219	10.1	2256.0	1	LT-HSC	2.3	84.1	296.3	92.7	46.0	41.1	yes
SRR13861937	LT-HSC6-55	20181226_batch1	20181227	13.6	1856.0	2	LT-HSC	5.3	85.6	297.9	94.7	45.0	29.4	yes
SRR13861936	LT-HSC6-56	20181226_batch1	20181227	10.6	1940.0	2	LT-HSC	5.1	87.6	297.7	94.5	48.5	44.6	yes
SRR13861935	LT-HSC6-57	20181226_batch1	20181227	12.2	1446.0	2	LT-HSC	3.4	87.5	298.1	94.6	46.0	37.6	yes
SRR13861934	LT-HSC6-58	20181226_batch1	20181227	12.1	1876.0	2	LT-HSC	3.4	90.2	297.1	93.8	46.0	36.3	yes
SRR13861887	LT-HSC6-59	20181226_batch1	20181227	11.1	1981.0	2	LT-HSC	4.6	88.5	297.9	95.4	46.0	39.4	yes
SRR13861885	LT-HSC6-60	20181226_batch1	20181227	5.2	1884.0	2	LT-HSC	4.0	87.4	297.6	94.2	48.0	40.1	yes
SRR13861884	LT-HSC6-61	20181226_batch2	20181227	6.8	1879.0	2	LT-HSC	4.3	87.5	297.9	94.8	49.0	40.8	yes
SRR13861883	LT-HSC6-62	20181226_batch2	20181227	10.3	1885.0	2	LT-HSC	3.7	87.7	297.8	94.8	48.0	41.3	yes
SRR13861882	LT-HSC6-63	20181226_batch2	20181227	7.1	1895.0	2	LT-HSC	4.3	88.0	297.3	94.5	49.0	42.5	yes
SRR13861881	LT-HSC6-64	20181226_batch2	20181227	7.3	1844.0	2	LT-HSC	5.4	86.7	297.7	95.0	48.0	43.5	yes
SRR13861880	LT-HSC6-65	20181226_batch2	20181227	8.5	1853.0	2	LT-HSC	3.5	87.7	297.7	94.8	48.0	40.8	yes
SRR13861879	LT-HSC6-66	20181226_batch2	20181227	10.2	1813.0	2	LT-HSC	4.0	87.2	297.7	94.9	48.0	40.7	yes
SRR13861878	LT-HSC6-67	20181226_batch2	20181227	7.6	1892.0	2	LT-HSC	3.5	86.2	297.1	95.0	48.5	42.8	yes
SRR13861876	LT-HSC6-68	20181226_batch2	20181227	8.7	1877.0	2	LT-HSC	4.8	85.7	296.7	94.8	48.0	44.7	yes
SRR13861875	LT-HSC6-69	20181226_batch2	20181227	5.1	1897.0	2	LT-HSC	3.8	87.3	298.2	95.1	47.0	37.9	yes
SRR13861873	LT-HSC6-70	20181226_batch2	20181227	11.1	1882.0	2	LT-HSC	3.5	88.6	297.5	94.5	48.0	44.2	yes
SRR13861872	LT-HSC6-71	20181226_batch2	20181227	10.7	1891.0	2	LT-HSC	3.4	87.6	297.5	94.9	48.0	42.7	yes
SRR13861855	LT-HSC6-72	20181226_batch2	20181227	8.8	1872.0	2	LT-HSC	3.2	87.3	297.5	94.7	49.0	44.9	no
SRR13861854	LT-HSC6-73	20181226_batch2	20181227	9.7	1867.0	2	LT-HSC	3.3	87.3	297.6	94.8	48.0	42.6	yes
SRR13861853	LT-HSC6-74	20181226_batch2	20181227	10.2	1839.0	2	LT-HSC	2.7	90.5	297.7	94.4	48.0	38.5	yes
SRR13861852	LT-HSC6-75	20181226_batch2	20181227	5.1	1873.0	2	LT-HSC	3.0	81.7	296.8	94.6	47.0	28.7	yes
SRR13861851	LT-HSC6-76	20181226_batch2	20181227	8.2	1866.0	3	LT-HSC	5.8	86.7	296.3	90.7	48.0	40.9	yes
SRR13861849	LT-HSC6-77	20181226_batch2	20181227	9.0	1860.0	3	LT-HSC	4.8	86.9	296.7	91.4	49.0	42.5	no
SRR13861848	LT-HSC6-79	20181226_batch3	20181227	12.4	1972.0	3	LT-HSC	6.0	89.0	297.5	91.1	45.0	31.8	yes
SRR13861846	LT-HSC6-81	20181226_batch3	20181227	8.7	1980.0	3	LT-HSC	5.6	87.7	297.1	91.4	46.0	32.1	yes
SRR13861845	LT-HSC6-82	20181226_batch3	20181227	11.0	2232.0	3	LT-HSC	5.7	89.0	297.6	92.0	47.0	37.1	yes
SRR13861844	LT-HSC6-83	20181226_batch3	20181227	10.6	1835.0	3	LT-HSC	4.9	90.2	297.3	91.1	48.0	40.3	yes
SRR13861843	LT-HSC6-84	20181226_batch3	20181227	12.5	2206.0	3	LT-HSC	4.4	89.5	297.4	91.4	46.0	33.8	yes
SRR13861842	LT-HSC6-85	20181226_batch3	20181227	11.9	1796.0	3	LT-HSC	4.5	88.4	297.7	91.7	47.0	39.3	yes
SRR13861841	LT-HSC6-86	20181226_batch3	20181227	4.6	1487.0	3	LT-HSC	4.6	67.3	296.8	91.7	45.0	40.6	yes
SRR13861840	LT-HSC6-87	20181226_batch3	20181227	10.8	1805.0	3	LT-HSC	6.3	87.6	297.3	91.5	45.0	28.6	yes
SRR13861822	LT-HSC6-88	20181226_batch3	20181227	12.0	1289.0	3	LT-HSC	5.0	85.9	297.2	91.9	46.0	29.9	yes
SRR13861820	LT-HSC6-90	20181226_batch3	20181227	18.1	1872.0	3	LT-HSC	5.6	90.2	297.6	91.9	47.0	38.3	yes
SRR13861819	LT-HSC6-91	20190108_batch1	20190109	15.6	2113.0	3	LT-HSC	4.9	89.8	297.6	91.6	47.0	36.3	yes
SRR13861818	LT-HSC6-92	20190108_batch1	20190109	11.6	1863.0	3	LT-HSC	5.0	90.3	297.4	91.5	49.0	42.6	yes
SRR13861817	LT-HSC6-93	20190108_batch1	20190109	12.2	2258.0	3	LT-HSC	4.8	89.6	297.6	91.2	47.0	37.1	yes
SRR13861816	LT-HSC6-94	20190108_batch1	20190109	15.1	2201.0	3	LT-HSC	5.1	88.5	297.7	91.8	47.0	39.5	yes
SRR13861815	LT-HSC6-95	20190108_batch1	20190109	12.7	1877.0	3	LT-HSC	5.1	80.8	297.5	91.5	45.0	31.6	yes
SRR13861814	LT-HSC6-96	20190108_batch1	20190109	8.0	1874.0	3	LT-HSC	5.8	87.7	297.1	91.2	47.0	38.5	yes
SRR13861813	LT-HSC6-97	20190108_batch1	20190109	8.9	1863.0	3	LT-HSC	5.9	88.0	297.1	91.5	48.0	39.5	yes
SRR13861811	LT-HSC6-98	20190108_batch1	20190109	8.0	1860.0	3	LT-HSC	5.3	87.2	297.2	92.0	48.0	42.3	yes
SRR13861810	LT-HSC6-99	20190108_batch1	20190109	6.7	1320.0	3	LT-HSC	4.7	82.1	296.3	91.1	48.0	42.2	yes
SRR13862091	LT-HSC6-100	20190108_batch1	20190109	9.6	1853.0	4	LT-HSC	4.0	85.0	297.4	94.9	48.0	41.8	yes
SRR13862080	LT-HSC6-101	20190108_batch1	20190109	8.1	1878.0	4	LT-HSC	3.2	84.9	296.7	94.6	48.0	44.5	yes
SRR13861970	LT-HSC6-102	20190108_batch1	20190109	9.2	1887.0	4	LT-HSC	3.3	86.3	297.6	95.1	47.0	43.5	yes
SRR13861870	LT-HSC6-103	20190108_batch1	20190109	8.3	1867.0	4	LT-HSC	3.4	86.5	297.1	94.7	48.0	42.7	yes
SRR13861858	LT-HSC6-104	20190108_batch1	20190109	11.5	1818.0	4	LT-HSC	3.0	84.9	297.1	94.8	47.0	39.3	yes
SRR13861832	LT-HSC6-105	20190108_batch1	20190109	8.5	1844.0	4	LT-HSC	4.1	86.4	297.6	95.2	48.0	45.4	no
SRR13861991	LT-HSC6-106	20190108_batch1	20190109	13.8	1851.0	4	LT-HSC	4.3	89.3	297.8	95.1	48.0	42.6	yes
SRR13861980	LT-HSC6-107	20190108_batch1	20190109	16.0	1865.0	4	LT-HSC	4.0	88.7	297.6	94.9	48.0	42.1	yes
SRR13861952	LT-HSC6-108	20190108_batch1	20190109	13.4	1835.0	4	LT-HSC	4.1	88.6	297.8	94.9	47.0	41.0	yes
SRR13861930	LT-HSC6-109	20190108_batch1	20190109	11.0	1865.0	4	LT-HSC	4.0	86.6	297.3	94.7	49.0	44.1	yes
SRR13862105	LT-HSC6-110	20190108_batch1	20190109	6.4	1863.0	4	LT-HSC	4.8	83.7	296.6	94.6	47.0	43.8	yes
SRR13862103	LT-HSC6-111	20190108_batch1	20190109	8.0	1857.0	4	LT-HSC	4.0	84.3	297.6	95.4	47.0	43.3	yes
SRR13862102	LT-HSC6-112	20190108_batch1	20190109	10.2	1425.0	4	LT-HSC	3.1	84.8	296.8	94.8	47.0	40.3	yes
SRR13862100	LT-HSC6-113	20190108_batch1	20190109	10.2	1833.0	4	LT-HSC	3.8	85.6	297.5	95.2	47.5	41.4	yes
SRR13862099	LT-HSC6-114	20190108_batch1	20190109	9.7	1836.0	4	LT-HSC	4.3	88.6	297.5	94.9	48.0	42.8	yes
SRR13862059	LT-HSC6-115	20190108_batch1	20190109	9.8	1856.0	4	LT-HSC	5.2	89.9	297.4	94.9	49.0	45.7	no
SRR13862058	LT-HSC6-116	20190108_batch1	20190109	11.2	1842.0	4	LT-HSC	3.4	85.5	297.3	95.0	48.0	44.9	yes
SRR13862057	LT-HSC6-117	20190108_batch1	20190109	12.6	1848.0	4	LT-HSC	3.2	87.2	297.4	95.0	47.0	41.1	yes
SRR13862056	LT-HSC6-118	20190108_batch1	20190109	16.1	1879.0	4	LT-HSC	3.7	88.3	297.5	95.1	48.0	41.2	yes
SRR13862055	LT-HSC6-119	20190108_batch1	20190109	13.1	1879.0	4	LT-HSC	3.7	87.1	298.1	95.2	50.0	43.3	yes
SRR13862053	LT-HSC6-120	20190108_batch1	20190109	14.9	1879.0	4	LT-HSC	4.0	92.0	298.2	95.2	48.0	41.9	yes
SRR13862052	LT-HSC6-121	20190108_batch1	20190109	11.8	1908.0	4	LT-HSC	3.9	89.2	297.9	95.1	48.0	40.1	yes
SRR13861809	ST-HSC7-1	20181211_batch1	20181212	3.8	1835.0	1	ST-HSC	4.3	86.0	297.8	91.7	51.0	42.2	yes
SRR13861764	ST-HSC7-2	20181211_batch1	20181212	8.2	1826.0	1	ST-HSC	6.0	89.8	297.9	92.0	49.0	42.2	yes
SRR13861753	ST-HSC7-3	20181211_batch1	20181212	8.6	1845.0	1	ST-HSC	4.6	90.2	298.2	92.8	49.0	41.8	yes
SRR13862072	ST-HSC7-4	20181211_batch1	20181212	8.1	1456.0	1	ST-HSC	5.0	82.8	297.2	88.2	51.0	43.9	yes
SRR13862042	ST-HSC7-5	20181211_batch1	20181212	9.6	1848.0	1	ST-HSC	6.6	89.4	297.9	91.8	51.0	41.2	yes
SRR13862030	ST-HSC7-6	20181211_batch1	20181212	3.9	1843.0	1	ST-HSC	2.6	89.3	297.3	90.2	51.0	43.6	yes
SRR13861917	ST-HSC7-7	20181211_batch1	20181212	7.6	1823.0	1	ST-HSC	5.1	87.6	298.0	92.7	51.0	45.1	yes
SRR13862007	ST-HSC7-8	20181211_batch1	20181212	8.0	1876.0	1	ST-HSC	3.0	89.2	298.2	92.5	49.0	35.7	yes
SRR13861996	ST-HSC7-9	20181211_batch1	20181212	11.3	1862.0	1	ST-HSC	5.3	89.8	298.1	92.9	49.0	42.3	yes
SRR13861808	ST-HSC7-10	20181211_batch1	20181212	5.7	1865.0	1	ST-HSC	3.3	90.0	297.8	92.2	50.0	41.7	yes
SRR13861796	ST-HSC7-11	20181211_batch1	20181212	16.8	1864.0	1	ST-HSC	3.7	89.5	297.3	92.2	48.0	40.6	yes
SRR13861772	ST-HSC7-12	20181211_batch1	20181212	14.0	1859.0	2	ST-HSC	3.6	92.4	298.7	94.7	49.0	41.3	yes
SRR13861771	ST-HSC7-13	20181211_batch1	20181212	16.4	1887.0	2	ST-HSC	3.7	88.9	298.3	94.6	49.0	42.1	yes
SRR13861770	ST-HSC7-14	20181211_batch1	20181212	12.9	1844.0	2	ST-HSC	3.5	92.7	298.3	94.3	49.0	41.6	yes
SRR13861769	ST-HSC7-15	20181211_batch1	20181212	8.5	1833.0	2	ST-HSC	3.5	89.0	298.6	95.0	49.0	38.3	yes
SRR13861768	ST-HSC7-16	20181211_batch1	20181212	5.0	1828.0	2	ST-HSC	3.4	90.8	298.4	94.8	49.0	38.2	yes
SRR13861767	ST-HSC7-17	20181211_batch1	20181212	14.7	1845.0	2	ST-HSC	4.0	86.0	298.4	95.0	50.0	42.8	yes
SRR13861766	ST-HSC7-18	20181211_batch1	20181212	8.8	1848.0	2	ST-HSC	4.3	92.4	298.6	94.8	50.0	45.0	yes
SRR13861765	ST-HSC7-19	20181211_batch1	20181212	12.1	1865.0	1	ST-HSC	4.7	90.5	297.9	92.2	48.0	41.5	yes
SRR13861762	ST-HSC7-20	20181211_batch1	20181212	17.1	1881.0	1	ST-HSC	4.5	90.7	297.5	89.9	50.0	44.9	yes
SRR13861761	ST-HSC7-21	20181211_batch1	20181212	16.6	1891.0	1	ST-HSC	5.3	92.1	297.8	92.2	49.5	45.6	yes
SRR13861760	ST-HSC7-23	20181211_batch1	20181212	8.7	1875.0	2	ST-HSC	3.2	83.6	298.6	94.8	50.0	44.5	yes
SRR13861759	ST-HSC7-24	20181211_batch1	20181212	6.8	1876.0	2	ST-HSC	4.2	84.3	298.8	94.8	50.0	42.6	yes
SRR13861758	ST-HSC7-25	20181211_batch1	20181212	16.0	1875.0	2	ST-HSC	3.8	91.7	298.4	94.9	49.0	46.8	yes
SRR13861757	ST-HSC7-26	20181211_batch1	20181212	11.6	1857.0	2	ST-HSC	3.2	90.2	298.7	95.2	49.5	44.2	yes
SRR13861756	ST-HSC7-27	20181211_batch1	20181212	16.2	1880.0	3	ST-HSC	5.6	90.7	297.4	91.6	50.0	47.6	no
SRR13861755	ST-HSC7-28	20181211_batch1	20181212	8.2	1867.0	3	ST-HSC	6.5	92.2	297.7	91.5	50.0	45.4	no
SRR13861754	ST-HSC7-29	20181211_batch1	20181212	9.8	1869.0	3	ST-HSC	6.3	88.4	297.9	91.8	50.0	44.6	yes
SRR13861751	ST-HSC7-30	20181211_batch1	20181212	4.2	1888.0	3	ST-HSC	7.0	57.4	297.7	91.1	53.0	30.5	yes
SRR13862098	ST-HSC7-31	20181211_batch1	20181212	11.8	1922.0	3	ST-HSC	5.4	87.2	297.7	91.6	49.0	37.4	yes
SRR13862097	ST-HSC7-32	20181211_batch1	20181212	7.7	1889.0	3	ST-HSC	6.6	90.3	297.7	91.2	49.0	39.9	yes
SRR13862096	ST-HSC7-33	20181211_batch1	20181212	15.7	1910.0	1	ST-HSC	5.1	91.0	298.0	92.8	48.0	42.5	yes
SRR13862095	ST-HSC7-34	20181211_batch1	20181212	9.2	1900.0	3	ST-HSC	3.3	86.1	297.7	91.3	49.0	40.6	yes
SRR13862094	ST-HSC7-35	20181218_batch1	20181219	13.3	1876.0	1	ST-HSC	5.3	88.9	297.2	92.2	49.0	41.7	yes
SRR13862093	ST-HSC7-36	20181218_batch1	20181219	11.2	1889.0	3	ST-HSC	4.2	87.6	297.9	91.9	49.0	32.2	yes
SRR13862092	ST-HSC7-37	20181218_batch1	20181219	10.8	1863.0	1	ST-HSC	5.5	89.0	297.6	92.2	48.0	39.4	yes
SRR13862075	ST-HSC7-38	20181218_batch1	20181219	11.5	1875.0	1	ST-HSC	3.9	91.3	297.5	92.1	48.0	41.9	yes
SRR13862074	ST-HSC7-39	20181218_batch1	20181219	8.5	1896.0	3	ST-HSC	1.9	88.2	297.5	91.7	49.0	35.3	yes
SRR13862071	ST-HSC7-40	20181218_batch2	20181219	10.1	1904.0	3	ST-HSC	5.4	87.0	296.4	90.9	49.0	46.2	yes
SRR13862070	ST-HSC7-41	20181218_batch2	20181219	10.5	1873.0	1	ST-HSC	5.1	88.3	297.3	92.2	48.0	38.8	yes
SRR13862069	ST-HSC7-42	20181218_batch2	20181219	15.3	1873.0	1	ST-HSC	4.4	90.6	298.0	92.1	48.0	37.6	yes
SRR13862068	ST-HSC7-43	20181218_batch2	20181219	14.0	1889.0	4	ST-HSC	3.6	90.7	298.2	95.5	50.0	41.2	yes
SRR13862067	ST-HSC7-44	20181218_batch2	20181219	8.7	1897.0	1	ST-HSC	4.8	89.8	297.5	92.4	48.5	37.6	yes
SRR13862066	ST-HSC7-45	20181218_batch2	20181219	9.8	1907.0	1	ST-HSC	5.7	89.6	297.6	92.4	49.0	41.0	yes
SRR13862065	ST-HSC7-46	20181226_batch1	20181227	12.6	1339.0	1	ST-HSC	5.7	85.3	296.9	92.3	46.0	33.4	yes
SRR13862064	ST-HSC7-47	20181226_batch1	20181227	13.8	1888.0	1	ST-HSC	3.4	90.7	297.5	92.3	47.0	39.6	yes
SRR13862063	ST-HSC7-48	20181226_batch1	20181227	11.6	1826.0	1	ST-HSC	3.7	85.4	297.3	92.1	46.0	34.6	yes
SRR13862060	ST-HSC7-49	20181226_batch1	20181227	11.8	1291.0	1	ST-HSC	3.5	86.3	297.4	92.2	46.0	38.6	yes
SRR13862041	ST-HSC7-50	20181226_batch1	20181227	7.1	1913.0	1	ST-HSC	3.9	85.3	296.5	91.9	46.0	29.0	yes
SRR13862040	ST-HSC7-51	20181226_batch1	20181227	7.2	1332.0	1	ST-HSC	5.6	87.6	296.6	91.5	47.0	40.0	yes
SRR13862039	ST-HSC7-52	20181226_batch1	20181227	9.2	1367.0	2	ST-HSC	3.6	87.7	297.1	94.4	46.5	36.7	yes
SRR13862038	ST-HSC7-53	20181226_batch1	20181227	9.7	1909.0	2	ST-HSC	3.1	89.9	297.9	94.6	48.0	44.2	yes
SRR13862037	ST-HSC7-54	20181226_batch1	20181227	8.8	1863.0	2	ST-HSC	3.5	88.5	297.9	94.7	47.0	38.0	yes
SRR13862036	ST-HSC7-55	20181226_batch1	20181227	7.2	1853.0	2	ST-HSC	4.2	86.9	298.2	94.6	46.0	31.7	yes
SRR13862035	ST-HSC7-56	20181226_batch1	20181227	7.2	1844.0	2	ST-HSC	4.2	84.9	298.3	94.7	47.0	43.6	yes
SRR13862034	ST-HSC7-57	20181226_batch2	20181227	9.4	1891.0	2	ST-HSC	4.6	89.1	298.3	94.9	47.0	38.2	yes
SRR13862032	ST-HSC7-58	20181226_batch2	20181227	5.3	1861.0	2	ST-HSC	2.4	79.2	293.4	89.0	46.0	42.0	yes
SRR13862031	ST-HSC7-59	20181226_batch2	20181227	1.8	1844.0	4	ST-HSC	4.5	85.9	298.0	94.6	48.0	41.7	yes
SRR13862029	ST-HSC7-61	20181226_batch2	20181227	5.4	1424.0	2	ST-HSC	3.1	84.2	297.6	93.9	45.0	24.9	yes
SRR13862028	ST-HSC7-62	20181226_batch2	20181227	9.4	1460.0	2	ST-HSC	3.7	83.3	298.6	94.8	44.5	25.2	yes
SRR13862027	ST-HSC7-63	20181226_batch2	20181227	8.1	1343.0	2	ST-HSC	2.8	80.4	298.4	94.4	45.0	32.9	yes
SRR13861923	ST-HSC7-65	20181226_batch2	20181227	10.9	1333.0	2	ST-HSC	2.1	87.3	296.5	93.8	46.0	42.2	yes
SRR13861922	ST-HSC7-66	20181226_batch2	20181227	3.0	1624.0	4	ST-HSC	3.8	2.2	295.5	93.4	49.0	44.2	no
SRR13861921	ST-HSC7-67	20181226_batch2	20181227	8.0	1876.0	4	ST-HSC	3.4	90.5	297.9	95.0	49.0	48.5	yes
SRR13861920	ST-HSC7-68	20181226_batch2	20181227	8.4	1875.0	4	ST-HSC	3.8	89.2	297.5	95.0	49.0	45.1	yes
SRR13861918	ST-HSC7-69	20181226_batch2	20181227	9.3	1838.0	4	ST-HSC	4.6	89.9	297.5	94.6	49.0	43.7	yes
SRR13861916	ST-HSC7-70	20181226_batch2	20181227	7.9	1857.0	2	ST-HSC	3.1	86.7	296.2	93.6	48.0	39.5	yes
SRR13861915	ST-HSC7-71	20181226_batch2	20181227	6.5	1856.0	4	ST-HSC	3.5	89.6	297.5	95.1	49.0	42.3	no
SRR13861914	ST-HSC7-72	20181226_batch3	20181227	8.4	1869.0	4	ST-HSC	5.1	88.4	297.7	95.0	48.0	41.6	yes
SRR13861913	ST-HSC7-73	20181226_batch3	20181227	8.2	1899.0	2	ST-HSC	3.5	89.0	297.6	94.4	49.0	44.4	yes
SRR13861912	ST-HSC7-74	20181226_batch3	20181227	12.1	1873.0	2	ST-HSC	3.9	91.6	298.4	94.6	49.0	44.8	yes
SRR13861911	ST-HSC7-75	20181226_batch3	20181227	8.8	1871.0	4	ST-HSC	4.7	88.1	296.7	94.6	49.0	49.0	no
SRR13861910	ST-HSC7-76	20181226_batch3	20181227	13.1	1866.0	2	ST-HSC	3.5	88.7	297.1	94.5	49.0	47.8	yes
SRR13861909	ST-HSC7-77	20181226_batch3	20181227	15.6	1919.0	2	ST-HSC	3.2	85.9	296.4	94.4	49.0	49.3	no
SRR13862009	ST-HSC7-78	20181226_batch3	20181227	12.2	1931.0	2	ST-HSC	3.2	87.2	296.6	94.4	48.5	49.1	no
SRR13862008	ST-HSC7-79	20181226_batch3	20181227	13.2	1945.0	3	ST-HSC	5.9	86.4	296.4	91.1	49.0	45.8	no
SRR13862006	ST-HSC7-80	20181226_batch3	20181227	11.6	1900.0	3	ST-HSC	5.1	85.0	295.9	91.0	48.0	43.8	no
SRR13862005	ST-HSC7-81	20181226_batch3	20181227	10.7	1901.0	3	ST-HSC	3.8	86.7	296.0	91.3	49.0	48.8	no
SRR13862004	ST-HSC7-83	20181226_batch3	20181227	9.0	1865.0	3	ST-HSC	5.2	85.5	296.9	89.8	47.0	34.8	yes
SRR13862003	ST-HSC7-84	20181226_batch3	20181227	12.8	1874.0	3	ST-HSC	3.3	88.0	297.9	91.9	49.0	45.1	yes
SRR13862002	ST-HSC7-85	20181226_batch3	20181227	10.1	1890.0	3	ST-HSC	2.2	82.1	298.2	94.2	47.0	32.9	yes
SRR13862001	ST-HSC7-86	20181226_batch3	20181227	11.9	1898.0	3	ST-HSC	3.3	86.3	297.1	91.0	46.0	33.4	yes
SRR13862000	ST-HSC7-87	20181226_batch3	20181227	11.0	1860.0	3	ST-HSC	3.4	83.8	297.5	91.4	46.0	34.8	yes
SRR13861998	ST-HSC7-88	20181226_batch3	20181227	12.8	1880.0	3	ST-HSC	3.5	87.6	297.1	92.0	47.0	37.2	yes
SRR13861997	ST-HSC7-89	20181226_batch3	20181227	7.7	1910.0	3	ST-HSC	2.7	87.1	296.4	90.7	48.0	43.5	yes
SRR13861995	ST-HSC7-90	20181226_batch3	20181227	3.3	2147.0	4	ST-HSC	4.3	85.3	297.9	95.2	46.0	35.0	yes
SRR13861994	ST-HSC7-91	20181226_batch3	20181227	14.0	1901.0	3	ST-HSC	4.4	89.6	297.7	91.7	46.0	34.8	yes
SRR13861791	ST-HSC7-92	20190108_batch1	20190109	11.0	1920.0	3	ST-HSC	2.9	88.3	297.2	91.2	49.0	42.8	no
SRR13861790	ST-HSC7-93	20190108_batch1	20190109	11.3	1890.0	4	ST-HSC	3.1	86.8	297.6	95.2	48.0	42.4	yes
SRR13861789	ST-HSC7-94	20190108_batch1	20190109	12.9	1891.0	3	ST-HSC	3.7	86.5	297.3	91.6	49.0	45.1	yes
SRR13861788	ST-HSC7-95	20190108_batch1	20190109	11.4	1906.0	3	ST-HSC	3.8	88.5	297.2	91.3	48.0	41.6	yes
SRR13861787	ST-HSC7-96	20190108_batch1	20190109	15.3	1878.0	3	ST-HSC	4.0	89.9	297.5	91.6	48.0	39.6	yes
SRR13861785	ST-HSC7-97	20190108_batch1	20190109	9.8	1883.0	3	ST-HSC	2.8	85.4	296.5	91.8	48.0	45.9	yes
SRR13861784	ST-HSC7-98	20190108_batch1	20190109	15.0	2436.0	3	ST-HSC	3.1	87.5	296.9	92.1	47.0	41.9	yes
SRR13861783	ST-HSC7-99	20190108_batch1	20190109	9.0	1901.0	4	ST-HSC	5.0	87.2	297.1	94.9	48.0	42.0	yes
SRR13861807	ST-HSC7-100	20190108_batch1	20190109	10.4	1892.0	4	ST-HSC	3.3	88.8	297.8	95.4	47.0	41.7	yes
SRR13861806	ST-HSC7-101	20190108_batch1	20190109	11.8	1912.0	4	ST-HSC	3.6	86.9	297.5	95.1	47.0	43.7	yes
SRR13861805	ST-HSC7-102	20190108_batch1	20190109	12.2	1923.0	4	ST-HSC	3.2	86.0	297.2	94.7	47.0	44.2	yes
SRR13861804	ST-HSC7-103	20190108_batch1	20190109	10.0	1865.0	4	ST-HSC	5.0	88.0	296.9	94.6	47.0	44.3	yes
SRR13861803	ST-HSC7-104	20190108_batch1	20190109	10.3	1875.0	4	ST-HSC	4.1	86.2	296.9	94.8	48.0	47.2	yes
SRR13861802	ST-HSC7-105	20190108_batch1	20190109	10.2	1872.0	4	ST-HSC	3.6	88.1	297.2	94.6	48.0	44.2	yes
SRR13861800	ST-HSC7-106	20190108_batch1	20190109	10.5	1865.0	4	ST-HSC	4.1	89.8	297.6	94.9	48.0	38.5	yes
SRR13861799	ST-HSC7-107	20190108_batch1	20190109	12.9	1848.0	4	ST-HSC	3.5	89.0	297.7	95.3	48.0	44.7	yes
SRR13861798	ST-HSC7-108	20190108_batch1	20190109	13.8	1862.0	4	ST-HSC	3.8	88.1	297.0	94.5	49.0	46.2	yes
SRR13861797	ST-HSC7-109	20190108_batch1	20190109	14.2	1863.0	4	ST-HSC	3.2	87.4	296.5	94.0	49.0	50.5	no
SRR13861795	ST-HSC7-110	20190108_batch1	20190109	12.0	1865.0	4	ST-HSC	3.4	85.8	296.5	94.0	47.0	44.5	yes
SRR13861794	ST-HSC7-111	20190108_batch1	20190109	13.4	1886.0	4	ST-HSC	4.6	88.3	297.6	95.0	48.0	43.7	yes
SRR13861793	ST-HSC7-112	20190108_batch1	20190109	10.3	1923.0	4	ST-HSC	4.2	89.6	297.2	94.7	49.0	47.8	no
SRR13861792	ST-HSC7-113	20190108_batch1	20190109	12.8	1891.0	4	ST-HSC	4.4	89.4	297.8	94.8	48.0	42.6	yes
SRR13861775	ST-HSC7-114	20190108_batch1	20190109	12.0	1910.0	4	ST-HSC	3.7	87.0	296.8	94.5	48.0	44.8	yes
SRR13861773	ST-HSC7-115	20190108_batch1	20190109	9.9	1852.0	4	ST-HSC	4.0	87.3	296.9	94.7	48.0	48.0	yes
SRR13861782	MPP8-1	20181211_batch1	20181212	12.4	1865.0	1	MPP	4.0	90.3	297.0	92.0	49.0	44.9	yes
SRR13861962	MPP8-2	20181211_batch1	20181212	17.5	1868.0	1	MPP	4.1	92.3	298.1	93.0	48.0	41.4	yes
SRR13861861	MPP8-3	20181211_batch1	20181212	11.7	1847.0	1	MPP	6.5	89.6	297.9	91.9	48.0	39.2	yes
SRR13861833	MPP8-4	20181211_batch1	20181212	16.3	1826.0	1	MPP	5.9	92.0	297.9	92.7	48.0	34.1	yes
SRR13861993	MPP8-5	20181211_batch1	20181212	14.2	1850.0	1	MPP	3.2	86.2	298.2	92.3	50.0	43.9	no
SRR13861982	MPP8-6	20181211_batch1	20181212	20.2	1894.0	1	MPP	3.4	92.7	297.9	91.9	48.0	41.4	yes
SRR13861954	MPP8-7	20181211_batch1	20181212	13.2	1892.0	1	MPP	4.5	89.8	298.2	92.5	49.0	37.7	yes
SRR13861931	MPP8-8	20181211_batch1	20181212	17.9	1882.0	1	MPP	7.3	89.9	298.1	92.2	49.0	32.4	yes
SRR13862116	MPP8-9	20181211_batch1	20181212	14.8	1887.0	1	MPP	6.2	90.4	298.1	92.2	48.0	37.2	yes
SRR13861781	MPP8-10	20181211_batch1	20181212	15.7	1859.0	1	MPP	4.6	91.9	297.7	91.9	49.0	36.3	yes
SRR13862085	MPP8-11	20181211_batch1	20181212	15.4	1863.0	1	MPP	4.7	90.5	298.0	92.8	48.0	36.0	yes
SRR13861974	MPP8-12	20181211_batch1	20181212	11.9	1861.0	1	MPP	5.0	89.4	297.4	91.9	48.0	38.8	yes
SRR13861969	MPP8-13	20181211_batch1	20181212	9.8	1864.0	1	MPP	4.7	88.6	297.7	92.2	48.0	38.2	yes
SRR13861968	MPP8-14	20181211_batch1	20181212	8.2	1857.0	1	MPP	6.0	89.6	298.0	91.4	49.0	42.1	yes
SRR13861967	MPP8-15	20181211_batch1	20181212	12.8	1851.0	1	MPP	2.6	91.6	297.5	91.7	48.0	45.0	yes
SRR13861966	MPP8-16	20181211_batch1	20181212	8.9	1816.0	1	MPP	4.1	88.3	297.6	92.0	47.0	37.5	yes
SRR13861965	MPP8-17	20181211_batch1	20181212	12.8	1870.0	1	MPP	4.8	89.8	298.1	92.4	48.0	39.5	yes
SRR13861964	MPP8-18	20181211_batch1	20181212	11.1	1889.0	1	MPP	4.8	87.5	298.1	93.1	46.0	40.3	yes
SRR13861963	MPP8-19	20181211_batch1	20181212	13.4	1875.0	1	MPP	4.2	89.5	297.8	91.8	48.0	39.4	yes
SRR13861961	MPP8-20	20181211_batch1	20181212	13.9	1871.0	1	MPP	3.3	84.8	298.0	92.1	49.0	38.5	yes
SRR13861871	MPP8-21	20181211_batch1	20181212	2.4	1874.0	1	MPP	4.8	89.8	298.3	92.5	48.0	32.9	yes
SRR13861869	MPP8-22	20181211_batch1	20181212	10.1	1857.0	1	MPP	4.6	90.8	297.5	92.5	48.0	35.8	yes
SRR13861868	MPP8-23	20181211_batch1	20181212	15.3	1835.0	1	MPP	5.3	68.2	298.0	92.5	47.0	39.4	yes
SRR13861867	MPP8-24	20181211_batch1	20181212	12.7	1808.0	2	MPP	3.3	91.5	298.5	94.9	49.0	42.9	yes
SRR13861866	MPP8-25	20181211_batch1	20181212	15.5	1851.0	1	MPP	5.6	93.3	298.1	92.2	49.0	31.2	yes
SRR13861865	MPP8-26	20181211_batch1	20181212	12.4	1831.0	2	MPP	3.4	88.3	298.5	94.8	48.0	36.7	yes
SRR13861864	MPP8-27	20181211_batch1	20181212	16.6	1857.0	2	MPP	4.1	90.4	298.3	94.9	49.0	44.3	yes
SRR13861863	MPP8-28	20181211_batch1	20181212	20.8	1834.0	1	MPP	4.1	89.6	297.7	92.2	48.0	42.5	yes
SRR13861862	MPP8-29	20181211_batch1	20181212	19.3	1848.0	1	MPP	5.3	90.8	298.2	92.5	49.0	43.8	yes
SRR13861860	MPP8-30	20181211_batch1	20181212	16.6	1832.0	1	MPP	4.9	88.6	298.2	92.6	49.0	44.9	yes
SRR13861976	MPP8-31	20181211_batch1	20181212	14.4	1804.0	2	MPP	3.8	89.7	298.6	95.5	49.0	40.0	yes
SRR13861857	MPP8-32	20181211_batch1	20181212	10.6	1814.0	1	MPP	4.0	90.8	297.9	91.8	48.0	38.4	yes
SRR13861856	MPP8-33	20181211_batch1	20181212	13.9	1825.0	2	MPP	3.5	90.5	298.5	95.0	48.5	41.7	yes
SRR13861839	MPP8-34	20181211_batch1	20181212	11.1	1811.0	1	MPP	4.9	90.4	298.3	92.9	48.0	38.1	no
SRR13861838	MPP8-35	20181211_batch1	20181212	9.4	1835.0	2	MPP	3.7	89.7	298.5	95.2	48.0	38.1	yes
SRR13861837	MPP8-36	20181211_batch1	20181212	10.6	1902.0	2	MPP	3.9	87.2	298.4	94.7	49.0	42.6	yes
SRR13861836	MPP8-37	20181211_batch1	20181212	4.4	1902.0	2	MPP	3.2	89.6	298.5	94.8	49.0	41.1	yes
SRR13861835	MPP8-38	20181211_batch1	20181212	12.5	1876.0	3	MPP	4.6	90.9	297.8	92.2	49.0	42.0	yes
SRR13861834	MPP8-39	20181211_batch1	20181212	10.7	1893.0	3	MPP	3.5	84.3	297.7	91.7	50.0	36.3	yes
SRR13861831	MPP8-40	20181211_batch1	20181212	15.4	1871.0	1	MPP	4.6	90.4	297.9	92.5	48.0	35.5	yes
SRR13861830	MPP8-41	20181211_batch1	20181212	16.8	1876.0	2	MPP	3.3	91.7	298.1	94.3	48.0	28.1	yes
SRR13861829	MPP8-42	20181211_batch1	20181212	10.4	1881.0	3	MPP	3.7	88.7	297.5	90.6	49.0	37.4	yes
SRR13861828	MPP8-45	20181218_batch1	20181219	10.8	1926.0	2	MPP	3.5	88.9	297.1	94.6	49.0	48.8	no
SRR13861827	MPP8-46	20181218_batch1	20181219	10.3	1878.0	2	MPP	3.1	87.4	297.0	94.5	47.0	37.1	yes
SRR13861826	MPP8-47	20181218_batch1	20181219	12.3	1753.0	2	MPP	2.7	87.7	297.8	94.6	47.0	33.9	yes
SRR13861825	MPP8-48	20181218_batch1	20181219	17.0	1867.0	3	MPP	4.4	90.9	297.7	92.2	48.0	38.3	yes
SRR13861824	MPP8-49	20181218_batch1	20181219	12.5	1875.0	3	MPP	3.9	88.0	296.8	91.7	49.0	43.2	yes
SRR13861992	MPP8-50	20181218_batch1	20181219	9.6	1889.0	3	MPP	2.9	89.4	297.1	91.4	49.0	42.6	yes
SRR13861990	MPP8-51	20181218_batch1	20181219	7.9	1829.0	2	MPP	3.6	89.3	297.4	94.4	48.0	41.6	yes
SRR13861989	MPP8-52	20181218_batch1	20181219	8.2	1850.0	2	MPP	1.8	90.1	298.0	95.0	49.0	42.0	yes
SRR13861988	MPP8-54	20181218_batch2	20181219	11.5	1812.0	2	MPP	2.0	89.5	297.8	95.1	46.5	35.7	yes
SRR13861987	MPP8-55	20181226_batch1	20181227	9.9	1833.0	2	MPP	3.6	88.5	298.3	95.1	46.5	40.7	yes
SRR13861986	MPP8-56	20181226_batch1	20181227	10.5	1345.0	2	MPP	3.6	88.1	297.5	94.3	47.5	42.6	yes
SRR13861985	MPP8-57	20181226_batch1	20181227	8.6	856.0	2	MPP	3.4	87.5	297.4	94.4	48.0	45.7	yes
SRR13861984	MPP8-58	20181226_batch1	20181227	9.5	1887.0	3	MPP	3.5	85.9	295.8	90.0	49.0	45.4	yes
SRR13861983	MPP8-59	20181226_batch2	20181227	9.9	1857.0	2	MPP	2.8	88.0	297.2	94.3	48.5	47.9	yes
SRR13861981	MPP8-60	20181226_batch2	20181227	9.7	1839.0	2	MPP	3.1	87.4	296.9	94.3	48.0	47.8	yes
SRR13861979	MPP8-61	20181226_batch2	20181227	6.6	1907.0	4	MPP	4.5	87.4	296.7	94.7	49.0	51.9	no
SRR13861978	MPP8-62	20181226_batch2	20181227	13.1	1908.0	2	MPP	4.0	85.8	296.6	94.3	49.0	46.5	yes
SRR13861960	MPP8-63	20181226_batch2	20181227	11.2	1868.0	2	MPP	3.4	87.7	297.3	94.3	49.0	48.1	yes
SRR13861959	MPP8-64	20181226_batch2	20181227	11.0	1888.0	2	MPP	3.6	86.1	296.9	94.7	49.0	48.1	yes
SRR13861958	MPP8-65	20181226_batch2	20181227	16.5	1859.0	2	MPP	4.1	89.7	297.9	94.7	48.0	41.2	yes
SRR13861957	MPP8-66	20181226_batch2	20181227	12.8	1865.0	4	MPP	3.6	90.5	298.1	95.3	49.0	45.1	yes
SRR13861956	MPP8-67	20181226_batch2	20181227	12.5	1869.0	4	MPP	6.1	89.5	297.5	94.8	49.0	46.3	no
SRR13861955	MPP8-68	20181226_batch2	20181227	2.6	1834.0	4	MPP	4.0	76.4	292.3	93.3	46.0	43.5	no
SRR13861953	MPP8-70	20181226_batch3	20181227	13.5	1858.0	2	MPP	3.1	88.5	297.6	94.8	47.0	39.4	yes
SRR13861951	MPP8-71	20181226_batch3	20181227	13.2	2029.0	2	MPP	3.2	88.4	297.5	94.5	47.0	38.3	yes
SRR13861950	MPP8-72	20181226_batch3	20181227	11.5	1876.0	2	MPP	4.5	85.8	297.3	94.3	46.0	35.9	yes
SRR13861949	MPP8-73	20181226_batch3	20181227	12.8	1894.0	2	MPP	4.0	85.5	298.3	95.1	46.0	29.9	yes
SRR13861948	MPP8-74	20181226_batch3	20181227	14.6	1911.0	2	MPP	2.5	88.0	297.9	94.7	47.0	36.4	yes
SRR13861947	MPP8-75	20181226_batch3	20181227	15.2	1961.0	2	MPP	3.7	88.9	298.2	94.7	48.0	42.8	yes
SRR13861946	MPP8-76	20181226_batch3	20181227	17.1	2337.0	3	MPP	3.2	89.4	297.3	91.4	47.0	36.2	yes
SRR13861945	MPP8-77	20181226_batch3	20181227	14.4	2157.0	3	MPP	3.5	87.6	296.6	91.0	47.0	40.8	yes
SRR13861933	MPP8-78	20181226_batch3	20181227	11.9	1531.0	3	MPP	2.8	85.1	296.1	90.8	47.0	37.4	yes
SRR13861932	MPP8-79	20181226_batch3	20181227	10.1	2291.0	3	MPP	3.1	81.9	295.3	90.4	46.0	35.3	yes
SRR13861929	MPP8-80	20181226_batch3	20181227	11.4	1813.0	3	MPP	2.5	87.1	297.2	91.6	47.0	36.1	yes
SRR13861928	MPP8-81	20181226_batch3	20181227	11.5	1928.0	3	MPP	2.7	86.2	297.2	91.7	47.0	36.9	yes
SRR13861927	MPP8-82	20181226_batch3	20181227	10.8	1870.0	3	MPP	3.4	86.7	297.1	91.6	47.0	32.6	yes
SRR13861926	MPP8-83	20190108_batch1	20190109	4.3	1787.0	3	MPP	2.9	79.8	296.0	91.0	48.0	43.2	yes
SRR13861925	MPP8-84	20190108_batch1	20190109	9.6	1379.0	3	MPP	2.9	84.1	296.2	91.3	47.0	41.9	yes
SRR13861924	MPP8-85	20190108_batch1	20190109	11.3	1426.0	3	MPP	3.8	84.3	296.4	92.0	47.0	42.1	yes
SRR13862121	MPP8-86	20190108_batch1	20190109	11.4	1372.0	3	MPP	3.2	85.5	296.2	90.6	46.0	37.0	yes
SRR13862120	MPP8-87	20190108_batch1	20190109	10.3	1849.0	3	MPP	2.7	85.5	296.2	91.1	48.0	42.8	yes
SRR13862119	MPP8-88	20190108_batch1	20190109	10.8	1379.0	3	MPP	3.6	80.7	295.4	90.5	47.0	42.9	yes
SRR13862118	MPP8-89	20190108_batch1	20190109	9.2	1364.0	3	MPP	3.6	81.9	296.2	91.3	47.0	37.9	yes
SRR13862115	MPP8-90	20190108_batch1	20190109	8.9	1830.0	3	MPP	3.9	85.4	296.6	91.3	47.0	40.0	yes
SRR13862113	MPP8-91	20190108_batch1	20190109	13.6	1874.0	3	MPP	4.0	87.0	297.3	91.2	48.0	41.1	yes
SRR13862112	MPP8-92	20190108_batch1	20190109	13.6	1832.0	3	MPP	3.6	87.0	296.9	91.1	47.0	39.3	yes
SRR13862111	MPP8-93	20190108_batch1	20190109	17.5	1870.0	3	MPP	3.5	86.0	297.6	91.4	47.0	38.2	yes
SRR13862110	MPP8-94	20190108_batch1	20190109	7.6	1875.0	3	MPP	3.9	86.8	297.2	91.3	47.0	39.8	yes
SRR13862109	MPP8-95	20190108_batch1	20190109	13.9	1881.0	3	MPP	2.8	87.7	297.0	92.0	48.0	44.3	yes
SRR13862108	MPP8-96	20190108_batch1	20190109	11.3	1889.0	3	MPP	3.0	85.4	296.4	91.0	48.0	42.3	yes
SRR13862107	MPP8-97	20190108_batch1	20190109	8.9	1492.0	3	MPP	4.3	82.1	296.0	91.3	46.0	33.7	yes
SRR13862106	MPP8-98	20190108_batch1	20190109	14.2	1756.0	4	MPP	6.2	87.4	297.7	94.6	46.0	38.5	yes
SRR13862104	MPP8-99	20190108_batch1	20190109	11.1	1884.0	4	MPP	7.2	86.8	297.2	94.6	50.0	46.1	no
SRR13861780	MPP8-100	20190108_batch1	20190109	9.1	1881.0	4	MPP	5.6	85.4	296.8	94.4	46.0	40.4	yes
SRR13861779	MPP8-101	20190108_batch1	20190109	9.2	1835.0	4	MPP	6.3	85.6	297.0	94.8	47.0	43.0	no
SRR13861778	MPP8-102	20190108_batch1	20190109	8.2	1842.0	4	MPP	5.7	85.6	296.6	94.7	48.0	46.2	no
SRR13861777	MPP8-103	20190108_batch1	20190109	9.1	1849.0	4	MPP	5.0	85.5	296.5	94.1	47.0	42.6	yes
SRR13861776	MPP8-104	20190108_batch1	20190109	11.0	1278.0	4	MPP	4.3	82.9	296.4	94.1	48.0	46.1	yes
SRR13862090	MPP8-105	20190108_batch1	20190109	10.0	1885.0	4	MPP	5.5	86.5	296.9	94.7	47.0	42.3	yes
SRR13862089	MPP8-106	20190108_batch1	20190109	7.9	1861.0	4	MPP	4.7	87.3	296.8	94.0	48.0	44.7	yes
SRR13862088	MPP8-107	20190108_batch1	20190109	10.8	991.0	4	MPP	5.9	87.7	297.2	94.8	48.0	43.4	yes
SRR13862087	MPP8-108	20190108_batch1	20190109	11.9	995.0	4	MPP	5.5	88.3	297.1	93.7	48.0	45.4	yes
SRR13862086	MPP8-109	20190108_batch1	20190109	17.2	993.0	4	MPP	7.3	91.7	297.6	93.5	47.0	41.6	no
SRR13862084	MPP8-110	20190108_batch1	20190109	12.0	989.0	4	MPP	6.1	90.0	297.8	94.6	47.0	42.5	yes
SRR13862083	MPP8-111	20190108_batch1	20190109	4.0	996.0	4	MPP	4.5	85.4	295.9	93.7	48.0	49.1	no
SRR13862082	MPP8-112	20190108_batch1	20190109	10.7	854.0	4	MPP	4.6	85.8	297.0	94.8	47.0	41.2	yes
SRR13862081	MPP8-113	20190108_batch1	20190109	9.9	986.0	4	MPP	5.2	88.0	297.2	94.7	48.0	45.2	yes
SRR13862079	MPP8-114	20190108_batch1	20190109	10.2	865.0	4	MPP	3.9	85.7	296.9	94.3	46.0	41.7	yes
SRR13862078	MPP8-115	20190108_batch1	20190109	7.7	835.0	4	MPP	5.6	82.4	296.3	94.0	46.0	38.4	yes
SRR13862077	MPP8-116	20190108_batch1	20190109	10.7	1956.0	4	MPP	5.0	85.8	297.1	94.8	47.0	45.9	yes
SRR13862076	MPP8-117	20190108_batch1	20190109	9.9	1857.0	4	MPP	6.3	86.9	297.2	94.3	46.0	38.7	yes
SRR13861977	MPP8-118	20190108_batch1	20190109	9.1	1446.0	4	MPP	5.1	86.9	297.3	94.7	47.0	41.0	yes
SRR13861975	MPP8-119	20190108_batch1	20190109	8.0	1420.0	4	MPP	5.2	86.7	297.3	95.0	47.0	42.2	yes
SRR13861973	MPP8-120	20190108_batch1	20190109	9.2	1424.0	4	MPP	6.0	87.7	297.4	95.4	47.0	42.7	yes
SRR13861972	MPP8-121	20190108_batch1	20190109	11.7	1860.0	4	MPP	5.0	91.5	298.1	94.9	49.0	40.4	yes
SRR13861971	MPP8-122	20190108_batch1	20190109	8.5	1869.0	4	MPP	4.5	89.2	297.5	95.1	48.0	42.8	yes

**Online-only Table 2:** Batch information and quality control for bulk short-read RNA-sequencing samples.

GEO Accession ID	Sample Name in GEO	Cell sorting batch	cDNA preparation date	Qubit concentration (ng/ul)	cDNA peak (bp)	Sequencing lane	Fastq file Label	Cell type	Total reads (million)	Uniquely mapped reads (%)	Average mapped length	Q30 (%)	GC (%)	TIN (median)
SRR13861944	p100_8 W_LT-HSC_1	20181212_batch1	20181213	7.9	1851	1	12-L4	LT-HSC	23.1	86.9	293.6	89.1	46.0	58.3
SRR13861888	p100_8W_LT-HSC_2	20181218_batch1	20181219	13.5	1905	3	18-BM1-L2	LT-HSC	22.2	85.6	291.1	86.3	47.0	62.3
SRR13861877	p100_8W_LT-HSC_3	20181218_batch2	20181219	16.6	1859	2	18-BM2-L1	LT-HSC	20.0	86.8	292.3	88.1	47.0	56.4
SRR13861850	p100_8W_LT-HSC_4	20181226_batch1	20181227	9.3	1421	4	26-BM1-L3	LT-HSC	17.6	85.2	292.9	88.6	46.0	55.1
SRR13861823	p100_8W_LT-HSC_5	20181226_batch2	20181227	7.4	1403	6	26-BM3-L1	LT-HSC	29.0	86.3	292.8	88.1	46.0	50.9
SRR13862073	p100_8W_ST-HSC_1	20181212_batch1	20181213	7.5	1530	1	12-S1	ST-HSC	22.6	87.4	294.0	89.7	46.0	54.5
SRR13862061	p100_8W_ST-HSC_2	20181218_batch1	20181219	14.0	1832	2	18-BM1-S2	ST-HSC	21.3	84.5	290.3	86.6	48.0	66.1
SRR13862033	p100_8W_ST-HSC_3	20181218_batch2	20181219	20.4	1893	3	18-BM2-S2	ST-HSC	24.8	85.3	292.6	84.9	47.0	60.3
SRR13861919	p100_8W_ST-HSC_4	20181226_batch1	20181227	11.1	1297	4	26-BM1-S2	ST-HSC	24.0	83.5	291.1	87.0	45.0	49.4
SRR13861908	p100_8W_ST-HSC_5	20181226_batch2	20181227	6.6	1383	6	26-BM3-S1	ST-HSC	26.3	84.8	291.5	87.2	45.5	51.4
SRR13861812	p100_8W_MPP_1	20181212_batch1	20181213	6.6	1298	1	12-M1	MPP	19.9	86.8	293.5	89.0	46.5	57.1
SRR13861801	p100_8W_MPP_2	20181218_batch1	20181219	8.9	1882	3	18-BM1-M2	MPP	18.4	85.3	291.3	87.3	47.0	62.2
SRR13861774	p100_8W_MPP_3	20181218_batch2	20181219	14.5	1908	2	18-BM2-M2	MPP	27.8	83.0	290.7	85.8	46.5	63.3
SRR13861763	p100_8W_MPP_4	20181226_batch1	20181227	8.7	1320	4	26-BM1-M1	MPP	24.7	83.8	291.9	88.2	45.0	52.5
SRR13861752	p100_8W_MPP_5	20181226_batch2	20181227	8.0	1361	6	26-BM3-M1	MPP	26.8	84.3	292.0	87.8	45.0	53.2

**Online-only Table 3:** Batch information and quality control for bulk long-read RNA-sequencing samples.

GEO Accession ID	Sample Name in GEO	cDNA preparation batch	No. of P100 samples mixed	Qubit concentration (ng/ul)	cDNA peak (bp)	Sequencing methods	Sample ID	Run	Cell type	Stage	Number Of Reads	Number Of Bases	Median Read Length	Mean Read Length	N50	Mean Quality	Median Quality	Mapped Reads (%)	Reads >Q7:
SRR13861890	P_8W_LT-HSC	20181213_batch1, 20181219_batch2, 20181227_batch3	16	133	1323	PacBio	8-6	P04DY19171604-1_r64031_20191206_091315_4_G02	LT-HSC	8W	239366	238119916	763	994.8	1541	47.2	37.3	88.52	239366 (100.0%) 238.1 Mb
SRR13861889	P_8 W_ST-HSC	20181213_batch1, 20181219_batch2, 20181227_batch3	14	149	1314	PacBio	8-7	P04DY19171605-1_r64031_20191206_091315_4_G02	ST-HSC	8W	245745	226569997	696	922	1406	47.4	37.4	88.13	245745 (100.0%) 226.6 Mb
SRR13862062	P_8 W_MPP	20181213_batch1, 20181219_batch2, 20181227_batch3	14	122	1324	PacBio	8-8	P04DY19171606-1_r64031_20191206_091315_4_G02	MPP	8W	250940	230891305	695	920.1	1398	48.1	37.7	88.98	250940 (100.0%) 230.9 Mb
SRR13861859	N_8 W_LT-HSC_1	20181219_batch1	1	19.5	1683	Nanopore	8-L1	V959-0101	LT-HSC	8W	6356335	7343750072	932	1155.3	1365	10.8	10.9	99.74	6355843 (100.0%) 7343.5 Mb
SRR13862101	N_8 W_LT-HSC_2	20181219_batch2	1	17.1	1859	Nanopore	8-L2	V959-S01-0101	LT-HSC	8W	2371682	2530358055	864	1066.9	1232	10.7	10.9	99.45	2303881 (97.1%) 2462.3 Mb
SRR13862051	N_8 W_ST-HSC_1	20181219_batch1	2	13.75	1820	Nanopore	8-S1	V959-S01-0102	ST-HSC	8W	3758835	3964994999	850	1054.8	1215	10.6	10.7	99.37	3625553 (96.5%) 3834.3 Mb
SRR13861905	N_8 W_ST-HSC_2	20181227_batch2	2	9.13	1380	Nanopore	8-S2	V959-S01-0103	ST-HSC	8W	2037766	1985569474	809	974.4	1081	10.6	10.7	99.2	1968031 (96.6%) 1921.3 Mb
SRR13861894	N_8 W_MPP_1	20181219_batch1	1	16	1832	Nanopore	8-M1	V959-S01-0104	MPP	8W	3685213	3561785473	783	966.5	1068	10.6	10.8	99.47	3570384 (96.9%) 3457.0 Mb
SRR13862019	N_8 W_MPP_3	20181227_batch2	1	12.5	1367	Nanopore	8-M3	V959-S01-0105	MPP	8W	2140511	1982333637	772	926.1	1006	9.9	9.9	99.2	2016327 (94.2%) 1873.6 Mb
